# Allergic bronchopulmonary mycosis caused by Penicillium luteum

**DOI:** 10.1016/j.mmcr.2016.11.007

**Published:** 2016-12-02

**Authors:** Chiyako Oshikata, Maiko Watanabe, Akemi Saito, Hiroshi Yasueda, Kazuo Akiyama, Yoichi Kamata, Naomi Tsurikisawa

**Affiliations:** a National Hospital Organization Saitama National Hospital, Department of Respirology, 2-1 Suwa, Wako, Saitama 351-0102, Japan; b National Hospital Organization Sagamihara National Hospital, Department of Allergy and Respirology, Sakuradai 18-1, Minami-ku, Sagamihara, Kanagawa 252-0392, Japan; c Division of Microbiology, National Institute of Health Science, 1-18-1 Kamiyoga, Setagaya-ku, Tokyo 158-8501, Japan; d National Hospital Organization, Sagamihara National Hospital, Clinical Research Center for Allergy and Rheumatology, 18-1 Sakuradai, Minami-ku, Sagamihara, Kanagawa 252-0392, Japan; e Faculty of Agriculture, Iwate University, 3-18-8 Ueda, Morioka, Iwate 020-8550, Japan

**Keywords:** Allergic bronchopulmonary mycosis, Aspergillus fumigatus, Penicillium, Bronchial asthma

## Abstract

A 65-year-old Japanese male had severe bronchial asthma had increased mold-containing sputum. Serum total IgE level had increased to 798 IU/mL and antigen-specific precipitating antibodies to P. luteum and P. notatum were present but not those reactive toward any species of Aspergillus. Chest computed tomography revealed central bronchiectasis and bronchial wall thickness. After antigen-specific provocation with 10 mg/mL of P. luteum, the patient developed asthma exacerbation, but not with A. fumigatus. We present a rare case of Penicillium-induced allergic bronchopulmonary mycosis caused by P. luteum.

## Highlights

• *Penicillium-*induced allergic bronchopulmonary mycosis caused by *P. luteum*
• Antigen-specific precipitating antibodies to *P. luteum* and *P. notatum* were present but not to any species of *Aspergillus*.
• Patient had experienced asthma exacerbation after provocation with of *P. luteum.*

## 1 Introduction

*Aspergillus fumigatus* is the most common cause of Allergic bronchopulmonary mycosis (ABPM). However, even though *Penicillium* species are among the most common fungi in the environment, ABPM due to *Penicillium* species is rare, accounting for only 1.9% of ABPM cases other than *Aspergillus fumigatus* [1]. In particular, only one case of *Penicillium*-associated ABPM for which the species was identified (*P. digitatum* or *P. rubrum*) has been reported [2]. Here, we present a rare case of *Penicillium*-associated ABPM, which was caused by *P. luteum*.

## 2 Case

A 65-year-old Japanese male ex-smoker (Pack-year; 58.5) reported having symptoms consistent with severe asthma since he was 36 years old. He had low lung function (forced expiratory volume in 1 s [%FEV1], 34.2%) and severe hyperresponsiveness to acetylcholine. The provocative acetylcholine concentration that yielded a 20% decrease in FEV1 was 0.197 mg/mL. In addition, he mounted immediate positive cutaneous reactions to mites, *Trichophyton* spp., and six different pollens, but not to *Aspergillus* or *Penicillium*. He required treatment for several asthma exacerbations annually despite receiving 900 μg of inhaled chlorofluorocarbon – beclomethasone dipropionate and 10 mg of oral prednisolone daily.

**Fig. 1:**
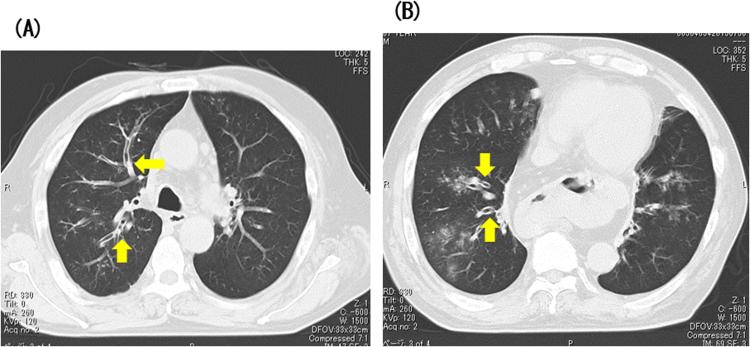
Computed tomography of the lung, performed at diagnosis. Computed tomography of the upper lung (A), and lower lung (B). Central bronchiectasis was present in right B3, B2 (A), and right B8, B9 (B) and 10 (arrows). Mucoid impaction was shown in right B3 (A) (arrows). Bronchial wall thickness was shown in right B8, B9 (B) and 10 (arrows).

When the patient was 54 years old, resection of nasal polyps followed by daily treatment with 1600 μg inhaled fluticasone propionate and 5 mg of prednisolone decreased the number of asthma exacerbations annually. At age 64 years (day 0), the patient had increased mold-containing sputum, the percentage of eosinophils as 13.8% (cells) and his serum total IgE level had increased from 259 IU/mL at age 39 years to 798 IU/mL. At this time (day 28), we again measured antigen-specific serum IgE levels as described and serum antigen-specific precipitating antibodies by Ouchterlony double immunodiffusion testing. Antigen of *P. chrysogenum* for measurement of IgE or *P. luteum* for precipitating antibodies was derived from Torii Pharmaceutical Co., Ltd, Tokyo, Japan. In contrast to his earlier results, the patient now had antigen-specific IgE antibodies to *Aspergillus* and *Penicillium*. In addition, antigen-specific precipitating antibodies to *P. luteum* and *P. notatum* were present but not those reactive toward any of 9 species of *Aspergillus. Penicillium* species was separated from mold-containing sputum at age 64 years, but more detailed species was not identified. Chest computed tomography revealed bronchial wall thickness, central bronchiectasis, and mucoid impaction (Fig. 1) (day 84).

**Fig. 2:**
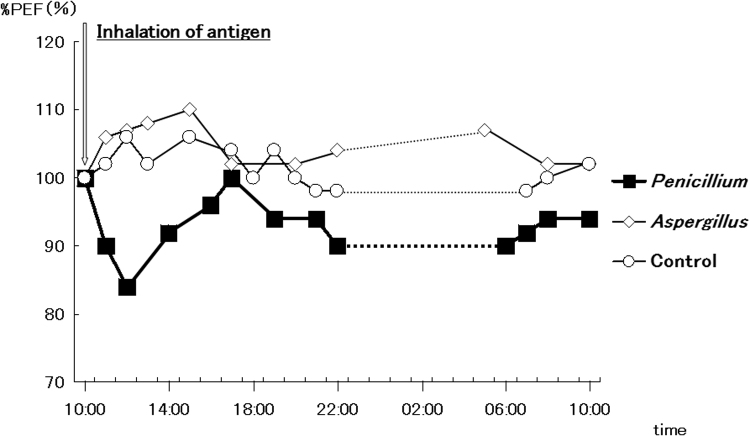
Results of bronchial provocation testing using *Penicillium luteum* or *Aspergillus fumigatus*. The patient was exposed to the indicated doses of *P. luteum* (solid squares) or *A. fumigatus* (open diamonds) or a negative control antigen (open circles), and the change in peak expiratory flow (%PEF) over in 1 min from the baseline value (100%) was recorded. A decrease of more than 15% (horizontal line) from baseline was defined as a positive reaction to the provocation protein fraction.

We obtained written informed consent from the patient to perform bronchial provocation tests using *P. luteum* and *A. fumigatus*. At bronchial provocation testing using *Penicillium l*10 min after antigen-specific provocation with 10 mg/mL of *P. luteum* (Torii Pharmaceutical Co., Ltd, Tokyo, Japan), the patient developed wheezing and chest tightness, and his peak expiratory flow decreased to 83.5% of that before antigen administration (day 98). He experienced a delayed hypersensitivity reaction 12 h after last provocation (day 99). However, inhalation of *A. fumigatus* (Torii Pharmaceutical Co., Ltd., Tokyo, Japan) did not elicit any changes in lung function or cause asthma exacerbation (Fig. 2) (day 105).

To collect particulates aerosolized from the patient's room or environment (day 133), we left open Petri dishes (plain coated with potato dextrose agar) plastic, 90×15 mm; SH90-15; Asahi Glass Co. Ltd., Tokyo, Japan) throughout the patient's bedroom for 10 min according to a report by Takatori et al. [3]. In addition, to collect airborne particulate matter, we distributed pieces of adhesive tape (Tegaderm Transparent Dressing 1625WJ; 6×7 cm; 3M Health Care, St Paul, MN) throughout his living spaces. And these samples by patient’ sputum were collected. These samples were cultured at 25 or 37 °C for several days; resulting colonies were identified by using morphologic evaluation and molecular methods. Specifically, partial sequences of the β-tubulin gene obtained by using the primers Bt2a and Bt2b were subjected to BLAST analysis.

To identify the antigen from the sputum for cultures, we added 1.5 mL of glass beads (Biospec Products, Bartlesville, OK) and homogenized the mixture by using a Mini-Beadbeater (Biospec Products). It was then incubated with 0.125 M NH_4_CO_3_ overnight at 4 °C; the antigen was extracted after freeze-drying of the filtrate. Samples cultured from the patient's bedroom, balcony, and parents’ house contained multiple species of *Penicillium*, but not *P. luteum* (Table I). However, the patient's asthma symptoms decreased after a healthy potted pothos plant in his living room was transplanted from mud into water (day 224); the mud from the cultivated pothos contained numerous colonies of diverse *Penicillium* species. In view of the collected data and observations, we diagnosed ABPM due to *P. luteum.*

## 3 Discussion

The genus *Penicillium* was first described more than 200 years ago [4], and since then, more than 1000 organisms have been assigned to it. A phylogenetic species concept has been used for *Penicillium* classification and identification and was facilitated by the incorporation of DNA sequencing techniques in the 1990s [5]. Both *Aspergillus* and *Penicillium* fungi typically are found indoors and are among the genera most frequently encountered in these environments. In addition, the high frequencies of identical residues and conservative substitutions between the amino acid sequences of *Aspergillus* and *Penicillium* [6] have led to inaccuracies and confusion regarding the taxonomy of these fungi. We did not examine precipitating antibodies to other *Penicillium* species than *P. luteum* or *P. notatum*. Furthermore, we performed bronchial provocation tests using *P. luteum,* but not *P. notatum*, and any other *Penicillium* species than *P. luteum*. Because we could not get commercially available antigens or not purified from environment or sputum. We considered the possibility for crossreactivity in *P. luteum* or *P. notatum* or other *Penicillium* species. We could not find *P. luteum* in his sputum or his living spaces. We consider *P. luteum* may be mixed in *Penicillium* species. Many kind species of *Penicillium* were resembling similar and it was difficult to identify each *Penicillium*. However, this patient exacerbated asthma after provocation of *P. luteum* but not *A. fumigatus*. We consider one of some causes for ABPM in this patient is due to *P. luteum*.

We elucidate a crossreactivity of *P. luteum* and *Penicillium* species in the future.

Here, we presented a rare case of *Penicillium-*specific ABPM, which was due to *P. luteum* and was not associated with *Aspergillus*, the most common cause of the disease. Despite our inability to isolate *P. luteum* from our patient's living spaces, we surmised that it was present among the *Penicillium* spp. in the soil of a potted pothos plant. Alternatively, the patient may have harbored antigen-specific antibodies for species closely related to *P. luteum* that may cross-react with *P. luteum* antigen. In this study, we isolated several strains (noted as “*Penicillium* spp.” in Table 1) that are morphologically or molecularly similar to *P. luteum*. The patient's sensitization to *Penicillium* but not *Aspergillus* may reflect his exposure to higher counts of, and more, *Penicillium* species compared with *Aspergillus* species.

**Table 1:** Molecular identification of *Penicillium* species from patient's sputum and living environment.

			*P. brevicompactum*	*P. citreonigrum*	*P. citrinum*	*P. glabrum*	*P. italicum*	*P. luteum*	*P. simplicissimum*	*P. steckii*	*Penicillium* spp.	*Talaromyces* spp.
											
Sputum						+	+				++	
											
Environment												
Bedroom									+		+	
Living room					+							
	soil of pothos plant				++						++	
Dining room									++			
Balcony					+	+				+		
	soil of Benjamin plant			+	+				+		++	
	soil of rosemary plant										+	
Parents’ house			+				+				+	++

+, present in one sample only; ++, present in 2 or more samples.

## Conflict of interest

The authors report no conflicts of interest in this work.

## References

[1] Chowdhary A., Agarwal K., Kathuria S., Gaur S.N., Randhawa H.S., Meis J.F. (2014). Allergic bronchopulmonary mycosis due to fungi other than *Aspergillus*: a global overview. Crit. Rev. Microbiol.

[2] Sahn S.A., Lakshminarayan S. (1973). Allergic bronchopulmonary penicilliosis. Chest.

[3] Takatori M., Shida T., Akiyama K., Takatori K. (1994). Airborne fungi during the last ten years in Sagamihara. Arerugi.

[4] H.F. Link, Observationes in Ordines plantarum naturales, Dissertatio 1ma, Magazin der Gesellschalt Naturforschenden Freunde Berlin 1809, 3, pp. 3–42

[5] Visagie C.M., Houbraken J., Frisvad J.C., Hong S.B., Klaassen C.H., Perrone G., Seifert K.A., Varga J., Yaguchi T., Samson R.A. (2014). Identification and nomenclature of the genus Penicillium. Stud. Mycol..

[6] Tan Z.B., Li J.F., Li X.T., Gu Y., Wu M.C., Wu J., Wang J.Q. (2014). A unique mono- and diacylglycerol lipase from *Penicillium cyclopium*: heterologous expression, biochemical characterization and molecular basis for its substrate selectivity. PLoS One.

